# Germ Cell Lineage Homeostasis in *Drosophila* Requires the Vasa RNA Helicase

**DOI:** 10.1534/genetics.119.302558

**Published:** 2019-09-04

**Authors:** Zeljko Durdevic, Anne Ephrussi

**Affiliations:** Developmental Biology Unit, European Molecular Biology Laboratory, Heidelberg D-69117, Germany

**Keywords:** **vas*a*, Vasa helicase, Chk2, piRNA pathway, germline, oogenesis

## Abstract

The conserved RNA helicase Vasa is required for germ cell development in many organisms. In *Drosophila melanogaster* loss of PIWI-interacting RNA pathway components, including Vasa, causes Chk2-dependent oogenesis arrest. However, whether the arrest is due to Chk2 signaling at a specific stage and whether continuous Chk2 signaling is required for the arrest is unknown. Here, we show that absence of Vasa during the germarial stages causes Chk2-dependent oogenesis arrest. Additionally, we report the age-dependent decline of the ovariole number both in flies lacking Vasa expression only in the germarium and in loss-of-function **vas*a* mutant flies. We show that Chk2 activation exclusively in the germarium is sufficient to interrupt oogenesis and to reduce ovariole number in aging flies. Once induced in the germarium, Chk2-mediated arrest of germ cell development cannot be overcome by restoration of Vasa or by downregulation of Chk2 in the arrested egg chambers. These findings, together with the identity of Vasa-associated proteins identified in this study, demonstrate an essential role of the helicase in the germ cell lineage maintenance and indicate a function of Vasa in germline stem cell homeostasis.

DEVELOPMENT of the *Drosophila* female gonad begins during the third larval instar with the formation of 16–25 somatic niches that will give rise to the future germaria (Panchal *et al.* 2017). Each germarium hosts germline stem cells (GSCs) that produce the germ cell lineage (Wieschaus and Szabad 1979). In adult females, germ cell development begins with the division of a GSC into a self-renewing stem cell and a differentiating daughter cell, the cystoblast (CB). The CB undergoes four rounds of mitosis with incomplete cytokinesis, such that a stage 1 egg chamber is ultimately composed of an oocyte and 15 nurse cells, surrounded by a layer of follicular epithelial cells [reviewed in Gilboa and Lehmann (2004)]. A newly formed egg chamber buds off from the germarium and joins a linear array of developing egg chambers to form an ovariole. Each *Drosophila* ovary consists of 16–25 ovarioles, corresponding to the number of germaria formed in the third-instar larva.

*Drosophila* oogenesis has been intensively studied and many genes found to regulate development of the germ cell lineage. Among the germline proteins essential for oogenesis is the conserved RNA helicase Vasa (Vas). Vas is expressed throughout oogenesis and localizes to the posterior pole of the oocyte and early embryo. In situations leading to absence of Vas from the oocyte posterior pole, germline and posterior patterning determinants fail to localize, germ (or pole) plasm does not form, and the resulting embryos lack posterior structures and primordial germ cells (Lasko and Ashburner 1988, 1990; Hay *et al.* 1990). In contrast to late oogenesis and embryos, little is known about the role of Vas during early oogenic stages. In early oogenesis **vas** has been implicated in the translational control of *mei-p26* and in regulation of GSC mitotic chromosome condensation (Liu *et al.* 2009; Pek and Kai 2011b). Complete absence of **vas** triggers oogenesis arrest induced by *checkpoint kinase 2* (*Chk2*) (Lasko and Ashburner 1990; Durdevic *et al.* 2018). Whether Chk2 is activated at a specific stage and whether continuous Chk2 signaling is required to arrest oogenesis has been unknown.

Using GFP-fused wild-type and trapping mutant (E400Q) Vas, we identified new Vas-associated proteins, several of which have a function in early germ cell development. To address the importance of Vas activity in early germ cell lineage development, we took a genetic approach. We found that, in addition to the previously described oogenesis arrest (Lasko and Ashburner 1988, 1990), loss-of-function **vas** mutation causes an age-dependent reduction of the number of egg chamber–producing ovarioles. Our study reveals that sole exclusion of Vas from the germarium causes Chk2-dependent arrest of oogenesis and a reduction of ovariole number in aging flies. Importantly, once induced in the germarium, Chk2-mediated oogenesis arrest and germline proliferation decline are not overcome by downregulation of Chk2 at later oogenic stages. Our study shows that Chk2 activity exclusively in the germarium is sufficient to interrupt germ cell development. Activity of Vas RNA helicase early in oogenesis is essential to prevent activation of Chk2 signaling and license the germline component of the *Drosophila* ovary for further development.

## Materials and Methods

### Fly stocks and husbandry

The following *Drosophila* stocks were used: *w^1118^*, *vas^1^ cn^1^ bw^1^/CyO* (*vas^PD^*, Schüpbach and Wieschaus 1986), b^1^ *vas*^*3*^*/CyO* (*vas^D1^*, Tearle and Nusslein-Volhard 1987; Lasko and Ashburner 1990), *P{w^+mC^* *=* *vas-GAL4.2.6}3/TM6B* (Kyoto *Drosophila* Genomics Resource Center: 109997), y^1^ w*; P{matα4-GAL-VP16}67; P{matα4-GAL-VP16}15 (*matTub-Gal4*, FBst0080361), *P{*w^+mC^ *=* *UASp-GFP.*vas*^WT^}VK00033/TM2 and P{*w^+mC^ *=* *UASp-GFP.*vas*^DQAD^}VK00033/TM2* (*GFP-*vas*^WT^* and *GFP-*vas*^DQAD^*, Xiol *et al.* 2014), *P{TRiP.GL00020}attP2/TM3* (TRiPmnk, FBst0035152), *P{TRiP.GL00094}attP2* (TRiPw, FBst0035573), and *P{Ubi-GFP.S65T}PAD1* (GFP, FBst0004888). All flies were kept at 25° on standard *Drosophila* medium.

### Generation of transgenic flies and expression of the transgenes

The **vas** transgene carrying the K295N substitution *(*vas*^GNT^)* was created by introducing the point mutation in wild-type **vas** complementary DNA sequence by site-directed mutagenesis using QuikChange II XL Site-Directed Mutagenesis Kit (Agilent). The transgene is subsequently cloned downstream of GFP into the pUASpK10attB vector (GenBank: EU729723.1, Koch *et al.* 2009), from which the K10 sequence was removed. Transgenes were integrated into the attP transposable element insertion site of the landing site line P{nos-phiC31\int.NLS}; ; PBac{y[+]-attP-3B}VK00033 (FBst0024871).

All transgenes are expressed using the GAL4/UAS-system. Gal4-drivers were under control of two promoters with distinct expression patterns: *vas*-Gal4 is expressed throughout oogenesis and matTub-Gal4 is excluded from the germarium (Supplemental Material, Figure S1B).

### Fecundity and hatching assays

Virgin females of *w^1118^* control and *vas^PD/D1^* and *vas^D1/D1^* genetic backgrounds with or without expressed transgenes were mated with *w^1118^* males for 24 hr at 25°. The crosses were then transferred to apple-juice agar plates, which were used to collect eggs at 24 hr intervals over 3 or 3–20 days. The number of laid eggs on each plate was counted and the plates were kept at 25° for 24 hr and the number of hatched larvae was also counted (Table S1 and Table S2). Experiments were performed in five independent replicates.

### Ovarian morphology and quantification of ovariole number

Ovaries were dissected from 3- to 20-day-old flies in PBS. To assess ovarian morphology, ovaries were directly imaged on Olympus SZX16 stereomicroscope. The length of the ovaries was measured using *Fiji* (Table S3, Table S4, and Table S5). For determination of egg chamber–producing ovariole number females were frozen and held at −20° before dissection. Ovaries were manually dissected under magnification in a drop of PBS. The ovarioles were gently separated from each other using wolfram needles. The ovariole number of each female was defined as a summary of the number of egg chamber–containing ovarioles in the right and left ovary (Table S6 and Table S7).

### Vas localization analysis

For Vas localization, ovaries from wild-type flies (*w*^*1118*^) and **vas** mutants (*vas^PD/D1^* and *vas^D1/D1^*) were fixed by incubation at 92° for 5 min in preheated fixation buffer (0.4% NaCl, 0.3% Triton X-100 in PBS), followed by extraction in 1% Triton X-100 for 1 hr at room temperature (RT). Fixed ovaries were incubated with primary antibodies Vas (rat; 1:500; Tomancak *et al.* 1998) and subsequently with secondary antibodies Alexa Fluor 647 conjugated donkey anti-rat IgG (1:1000; Jackson ImmunoResearch). Ovaries from flies expressing fusion proteins were fixed in 2% paraformaldehyde and 0.01% Triton X-100 for 15 min at RT. Fixed ovaries were mounted on glass slides for examination of GFP fluorescence for the fusion proteins and Alexa Fluor 647 fluorescence for wild-type Vas using a Zeiss LSM 780 confocal microscope. Nuclei were visualized with DAPI.

### Cuticle preparation

To examine larval cuticles, eggs were allowed to develop fully for 24 hr at 25°, dechorionated in bleach, and then transferred to a microscope slide bearing a drop of Hoyer’s medium mixed 1:1 with lactic acid. Cuticle preparations were heated at 65° overnight before examination using Zeiss Axiophot microscope. The number of counted larvae with or without abdomen is represented in Table S8.

### Immunohistochemical staining of ovaries and embryos

Freshly hatched females were mated with wild-type males and kept for 2–3 days on yeast at 25° before dissection. Ovaries were dissected in PBS and immediately fixed by incubation at 92° for 5 min in preheated fixation buffer (0.4% NaCl, 0.3% Triton X-100 in PBS), followed by extraction in 1% Triton X-100 for 1 hr at RT. Fixed ovaries were incubated with primary antibodies Aub (rabbit; 1:500; Homolka *et al.* 2015), Ago3 (mouse; 1:250; Gunawardane *et al.* 2007), and Vas (rat; 1:500; Tomancak *et al.* 1998). The following secondary antibodies were used: Alexa Fluor 488 conjugated goat anti-rabbit (1:1000; Invitrogen, Carlsbad, CA), anti-mouse IgG (1:500; Invitrogen), and Alexa Fluor 647 conjugated donkey anti-rat IgG (1:1000; Jackson ImmunoResearch). Nuclei were stained with DAPI.

For embryo staining, freshly hatched females were mated with wild-type males and fed with yeast for 2–3 days at 25° before egg collection. Embryos (0–1 or 1–3 hr) were collected and dechorionated in 50% bleach, then fixed by incubation at 92° for 30 sec in preheated fixation buffer (0.4% NaCl, 0.3% Triton X-100 in PBS), followed by devitellinization by rigorous shaking in a 1:1 mix of heptane and methanol. After washing in 0.1% Tween-20, embryos were either immediately incubated with primary antibodies against Aub (rabbit; 1:500; Homolka *et al.* 2015), Ago3 (mouse; 1:250; Gunawardane *et al.* 2007), and Vas (rat; 1:500; Tomancak *et al.* 1998), or stored in methanol at −20° for staining later on. The following secondary antibodies were used: Alexa Fluor 488 conjugated goat anti-rabbit (1:1000; Invitrogen), anti-mouse IgG (1:500; Invitrogen), and Alexa Fluor 647 conjugated donkey anti-rat IgG (1:1000; Jackson ImmunoResearch). Nuclei were stained with DAPI.

The samples were observed using a Zeiss LSM 780 or Leica SP8 confocal microscope. Oocytes and embryos with Aub- and Ago3-positive pole plasm were counted in three independent replicates (Table S9 and Table S10).

### Protein extraction and Western blotting

For whole protein lysates of ovaries, ∼20 pairs of ovaries from 3- to 5-day-old flies were homogenized in protein extraction buffer [25 mM Tris, pH 8.0, 27.5 mM NaCl, 20 mM KCl, 25 mM sucrose, 10 mM EDTA, 10 mM EGTA, 1 mM DTT, 10% (v/v) glycerol, 0.5% NP40, 1% Triton X-100, 1× Protease Inhibitor Cocktail; Roche]. For whole protein embryo lysates, 0–2 or 1–3 hr old embryos were collected from apple-juice agar plates and homogenized in protein extraction buffer. Samples were incubated on ice for 10 min, followed by two centrifugations, each 15 min at 16.000 × *g*. Then, 50–100 μg of total protein extracts were solubilized in SDS sample buffer by boiling at 95° for 5 min and analyzed by SDS-PAGE (4–12% NuPAGE gel; Invitrogen). Western blotting was performed using antibodies against Vas (rat, 1:3000; Tomancak *et al.* 1998) and Tub (mouse, 1:10,000, T5168; Sigma, St. Louis, MO). The following secondary antibodies were used: horseradish peroxidase (HRP)–conjugated goat anti-rat (1:10,000; GE Healthcare) and HRP-conjugated sheep anti-mouse IgG (1:10,000; GE Healthcare).

Quantification of relative protein expression levels was performed using ImageJ. A frame was placed around the most prominent band on the image and used as a reference to measure the mean gray value of all other protein bands, as well as the background. Next, the inverted value of the pixel density was calculated for all measurements by deducting the measured value from the maximal pixel value. The net value of target proteins and the loading control was calculated by deducting the inverted background from the inverted protein value. The ratio of the net value of the target protein and the corresponding loading control represents the relative expression level of the target protein. Fold-change was calculated as the ratio of the relative expression level of the target protein in the wild-type control over that of a specific sample.

### Protein immunoprecipitation and proteomic analysis

For protein immunoprecipitation, ovaries of 3- to 5-day-old *vas^PD/D1^*; *vas-Gal4 > GFP-*vas*^WT^*, *vas^PD/D1^*; *vas-Gal4 > GFP-*vas*^DQAD^* and control *Act5C-Gal4/CyO*; *Ubi-GFP* flies were dissected in PBS and homogenized in protein extraction buffer [25 mM Tris, pH 8.0, 27.5 mM NaCl, 20 mM KCl, 25 mM sucrose, 10 mM EDTA, 10 mM EGTA, 1 mM DTT, 10% (v/v) glycerol, 0.5% NP40, 1% Triton X-100, 1× Protease Inhibitor Cocktail; Roche]. After 10 min incubation on ice an equal volume of protein extraction buffer without detergents was added to the samples, and the samples were centrifuged twice for 15 min at 16,000 × *g*. Immunoprecipitations were performed using GFP-trap magnetic agarose beads (ChromoTek) at 4° for 1 hr on 20 mg of protein lysates. The beads were washed five times for 5 min at 4° in 1× in protein extraction buffer, then 1× in high-salt buffer (25 mM Tris, pH 8.0, 1 M NaCl, 0.5% NP40, 1% Triton X-100, 1× Protease Inhibitor Cocktail; Roche), then in 1× medium salt buffer (25 mM Tris, pH 8.0, 0.5 M NaCl, 0.5% NP40, 1% Triton X-100, 1× Protease Inhibitor Cocktail; Roche), then in 1× low-salt buffer (25 mM Tris, pH 8.0, 150 mM NaCl, 0.5% NP40, 1% Triton X-100, 1× Protease Inhibitor Cocktail; Roche), and finally, in 1× low-salt buffer without detergents (25 mM Tris, pH 8.0, 150 mM NaCl, 1× Protease Inhibitor Cocktail; Roche). After washing, precipitated proteins were eluted from the magnetic beads in elution buffer (200 mM glycine, pH 2.5) and neutralized with 1/10 volume of 1 M Tris base (pH 10.4).

Proteomic experiments were performed as described in Casabona *et al.* (2013). In brief, three samples were stacked in the SDS polyacrylamide gel and after Coomassie staining each lane was cut into three blocks, which were processed separately. After digestion with trypsin (sequencing grade; Promega, Madison, WI), the resulting peptides were analyzed by liquid chromatography with tandem mass spectrometry (LTQ-Orbitrap Velos pro; Thermo Fisher Scientific). Peptides and proteins from each mass spectrometry run were identified using Scaffold software and results for each lane were displayed. The selection criteria for the displayed proteins were as follows: a minimum of two peptides per protein should be identified and the peptide Mascot score should be at least 20. The experiment was performed in two biological replicates and specific interaction partners were determined by statistical analysis of control and positive samples using extracted spectral counts. A protein was considered as a high-confidence binding partner if its enrichment was ≥2 and the *P*-value was ≤0.05 in positive immunoprecipitations compared to controls. All proteins that showed enrichment ≥2 but had a higher *P*-value were considered low-confidence hits. *P*-values were computed using the web-based Quantitative Proteomics *P*-value Calculator (Chen *et al.* 2014), which applies a distribution-free permutation method based on simulation of the log(ratio). A pseudocount of 1 was used in all samples for proteins with no spectral counts. Unweighted spectrum counts for both replicates and the results of the statistical analysis are provided in Table S11. Analyses of gene ontology enrichment of biological processes and cellular components (Table S11) are based on Bioconductor package *clusterProfiler* (https://bioconductor.org/packages/release/bioc/html/clusterProfiler.html).

To validate the association of Vas with proteins identified in our proteomic analysis, immunoprecipitation of GFP and GFP-Vas^DQAD^ was performed as described above, using 30 mg of protein lysates. Co-immunoprecipitated proteins were detected by Western blotting using antibodies directed against Armi (goat; 1:500; Santa Cruz Biotechnology), Aub (rabbit; 1:1000; Homolka *et al.* 2015), Bel (rabbit; 1:500; Pek and Kai 2011a), GFP (rabbit; 1:5000; Chemokine), Nop60B (rabbit; 1:500; Riccardo *et al.* 2007), PABP (rabbit; 1:5000; kind gift from Matthias Hentze), Rm62 (rabbit; 1:250; Lei and Corces 2006), and Tub (mouse; 1:10,000, T5168; Sigma). The following secondary antibodies were used: HRP-conjugated donkey anti-rabbit (1:10,000; GE Healthcare), HRP-conjugated sheep anti-mouse IgG (1:10,000; GE Healthcare), and HRP-conjugated rabbit anti-goat IgG (1:10,000; Sigma). To reprobe Western blot membranes with another antibody, we removed bound primary and secondary antibodies from membranes using Restore PLUS Western reagent (Thermo Scientific) according to the manufacturer’s recommendations.

### Fluorescence *in situ* RNA hybridization

Fluorescence *in situ* RNA hybridization experiments were performed as described in Gaspar *et al.* (2017). In brief, ovaries were dissected in PBS and immediately fixed in 2% paraformaldehyde and 0.05% Triton X-100 in PBS for 20 min at RT. After washing in PBT (PBS plus 0.1% Triton X-100) samples were treated with 2 µg/ml proteinase K in PBT for 5 min and then were subjected to 95° in PBS plus 0.05% SDS for 5 min. Samples were prehybridized in 200 µl hybridization buffer (300 mM NaCl, 30 mM sodium citrate, pH 7.0, 15% ethylene carbonate, 1 mM EDTA, 50 µg/ml heparin, 100 µg/ml salmon sperm DNA, 1% Triton X-100) for 10 min at 42°. Fluorescence-labeled oligonucleotides (12.5–25 nM) were prewarmed in hybridization buffer and added to the samples. Hybridization was allowed to proceed for 2 hr at 42°. Samples were washed three times for 10 min at 42° in prewarmed buffers (1× hybridization buffer, then 1× hybridization buffer:PBT in a 1:1 mixture, followed by 1× PBT). The final washing step was performed in prewarmed PBT at RT for 10 min. The samples were mounted in 80% 2,2-thiodiethanol in PBS and analyzed on a Leica SP8 confocal microscope.

### Labeling of DNA oligonucleotides for fluorescence *in situ* RNA hybridization

Labeling of the oligonucleotides was performed as described in Gaspar *et al.* (2017). Briefly, nonoverlapping arrays of 18–22 nt long DNA oligonucleotides complementary to *mnk* (Table S12) were selected using the smFISHprobe_finder.R script (Gaspar *et al.* 2017). An equimolar mixture of oligos for a given RNA was fluorescence-labeled with Alexa Fluor 565– or Alexa Fluor 633–labeled ddUTP using terminal deoxynucleotidyl transferase. After ethanol precipitation and washing with 80% ethanol, fluorescence-labeled oligonucleotides were reconstituted with nuclease-free water.

### RNA extraction and quantitative PCR analysis

Total RNA was extracted from ovaries of 3-day-old flies using TRIzol reagent (Thermo Fisher Scientific). For first-strand complementary DNA synthesis, RNA was reverse-transcribed using the QuantiTect Reverse Transcription Kit (QIAGEN, Valencia, CA). Quantitative PCR was performed on a StepOne Real-Time PCR System (Thermo Fisher Scientific) using SYBR Green PCR Master Mix (Thermo Fisher Scientific). Experiments were performed in biological triplicates with technical triplicates. Relative RNA levels were calculated by the 2^−ΔΔCT^ method (Livak and Schmittgen 2001), normalized to *rp49* mRNA levels and normalized to respective RNA levels from *w^1118^* flies. Sequences of primers used for quantitative PCR reaction are presented in Table S12.

### Data availability

The authors declare that all data supporting the findings of this study are available within the manuscript and its supplemental files. Supplemental material available at FigShare: https://doi.org/10.25386/genetics.9026579.

## Results

### Vas helicase activity is required for *Drosophila* oogenesis

To investigate effects of Vas helicase activity on germ cell lineage development, we used the UAS/GAL4 system to manipulate the expression of wild-type Vas and of two Vas helicase mutants. The Vas mutant proteins contain amino acid substitutions that affect helicase activity at different points in the enzymatic process: the K295N (GKT→GNT) substitution hinders ATP and RNA binding and locks the helicase in an open conformation, whereas the E400Q (DEAD→DQAD) mutation prevents release of the ATP-hydrolysis products and locks the helicase in a closed conformation (Xiol *et al.* 2014) (Figure S1A). To monitor expression and localization of the different Vas proteins, we fused them with GFP (GFP-Vas^DQAD^, GFP-Vas^GNT^, and GFP-Vas^WT^).

**Figure 1:**
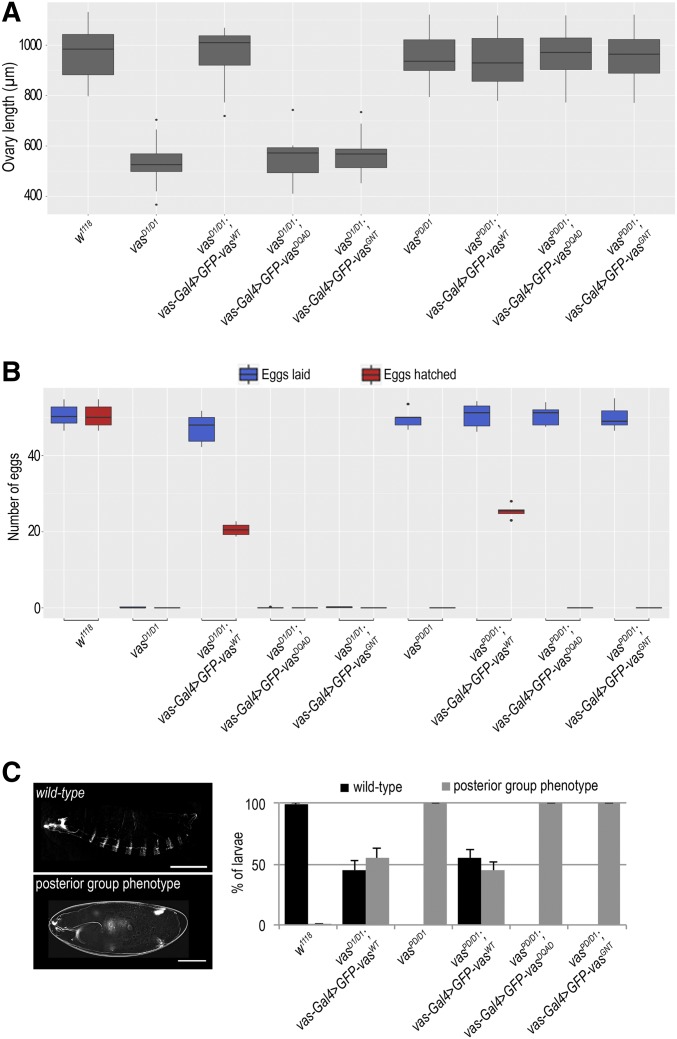
Vasa helicase activity is essential for germline and embryo development. (A) Box plot representing length of ovaries of wild-type (*w^1118^)*, *vas^D1/D1^*, *vas^D1/D1^*; *vas-Gal4 > GFP-Vas^WT^*, *vas^D1/D1^*; *vas-Gal4 > GFP-Vas^DQAD^*, *vas^D1/D1^*; **vas*-Gal4 > GFP-Vas^GNT^*, *vas^PD/D1^*, *vas^PD/D1^*; *vas-Gal4 > GFP-Vas^WT^*, *vas^PD/D1^*; *vas-Gal4 > GFP-Vas^DQAD^*, and *vas^PD/D1^*; *vas-Gal4 > GFP-Vas^GNT^* flies. The measurements were performed on 10 flies (*n* = 10) of each genotype. Dots in the box plot represent values that are 1.5 times greater than the upper limit or 1.5 time smaller than the lower limit of the interquartile range. (B) Box plot representing the number of eggs laid and hatched from wild-type (*w^1118^)*, *vas^D1/D1^*, *vas^D1/D1^*; *vas-Gal4 > GFP-Vas^WT^*, *vas^D1/D1^*; *vas-Gal4 > GFP-Vas^DQAD^*, *vas^D1/D1^*; *vas-Gal4 > GFP-Vas^GNT^*, *vas^D1/D1^*, *vas^D1/D1^*; *vas-Gal4 > GFP-Vas^WT^*, *vas^D1/D1^*; *vas-Gal4 > GFP-Vas^DQAD^*, and *vas^D1/D1^*; *vas-Gal4 > GFP-Vas^GNT^* flies. Five independent replicates of the experiment were performed. Dots in the box plot represent values that are 1.5 times greater than the upper limit or 1.5 time smaller than the lower limit of the interquartile range. (C) Larval cuticle phenotypes observed in wild-type (*w^1118^)*, *vas^D1/D1^*; *vas-Gal4 > GFP-Vas^WT^*, in *vas^D1/D1^*, *vas^PD/D1^*; *vas-Gal4 > GFP-Vas^WT^*, *vas^PD/D1^*; *vas-Gal4 > GFP-Vas^DQAD^*, and *vas^PD/D1^*; *vas-Gal4 > GFP-Vas^GNT^* flies. Bars, 500 µm (larva) and 100 µm (unhatched larva).

We tested the ability of the proteins to provide Vas function and suppress the oogenesis arrest displayed by *vas^D1/D1^* mutants and rescue the abdominal defects (posterior group phenotype) of embryos produced by the hypomorphic *vas^PD/D1^* females. We assessed ovarian morphology and quantified ovary length as a measure of oogenesis rescue (Figure S1C). The GFP-Vas^WT^ transgene fully restored oogenesis to the loss of function *vas^D1/D1^* flies, whereas the helicase mutants GFP-Vas^DQAD^ and GFP-Vas^GNT^ did not (Figure 1A and Figure S1D). Furthermore, Vas function provided by the GFP-Vas^WT^ transgene promoted abdomen formation in ∼50% of embryos produced by both *vas^D1/D1^* and *vas^PD/D1^* females (Figure 1, B and C). In contrast, the embryos produced by helicase inactive GFP-Vas^DQAD^ and GFP-Vas^GNT^ expressing *vas^PD/D1^* females had a strong posterior group phenotype and did not hatch (Figure 1, B and C). These results suggest that the helicase activity of Vas is required for oogenesis and embryogenesis.

### Localization of PIWI proteins is affected by Vas’s helicase activity

Localization of Vas in the egg chamber is independent of RNA-binding and helicase activity (Liang *et al.* 1994; Dehghani and Lasko 2016). We analyzed whether the E400Q and K295N mutations (Figure S1A), which impair Vas helicase activity differently, locking the enzyme in a closed or in an open conformation, respectively, affect localization of the protein. Additionally, as Aub and Ago3 colocalize with Vas (Liang *et al.* 1994; Malone *et al.* 2009), we tested if the localization of these two PIWI proteins in the egg chamber and embryo is affected by Vas helicase mutations.

**Figure 2:**
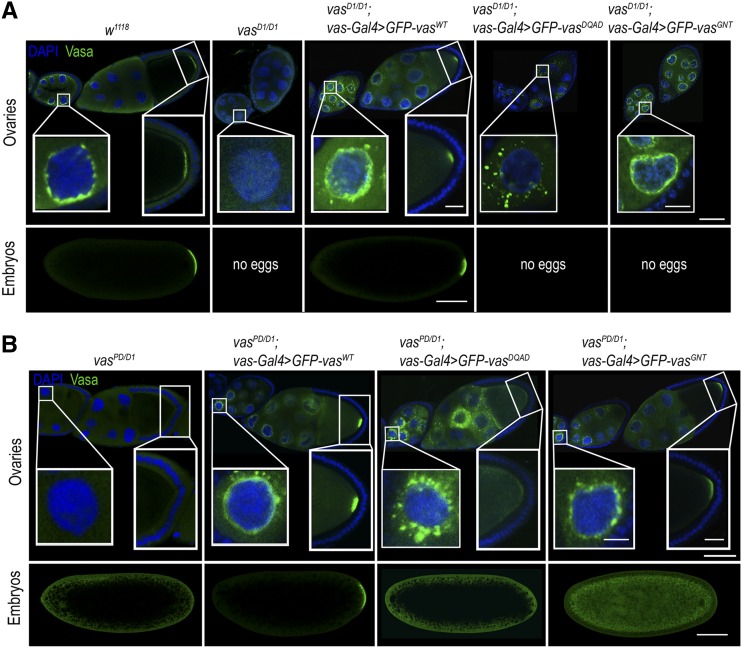
Helicase activity is required for Vasa localization at the posterior pole of the embryo. (A) Localization of Vasa in egg chambers (top panels) and embryos (bottom panels) of wild-type (*w^1118^)*, *vas^D1/D1^*, *vas^D1/D1^*; *vas-Gal4 > GFP-Vas^WT^*, *vas^D1/D1^*; *vas-Gal4 > GFP-Vas^DQAD^*, and *vas^D1/D1^*; *vas-Gal4 > GFP-Vas^GNT^* flies. Bars, 50 µm (egg chamber), 10 µm (nuage and pole plasm), and 100 µm (embryo). (B) Localization of Vasa in egg chambers (top panels) and embryos (bottom panels) of *vas^PD/D1^*, *vas^PD/D1^*; *vas-Gal4 > GFP-Vas^WT^*, *vas^PD/D1^*; *vas-Gal4 > GFP-Vas^DQAD^*, and *vas^PD/D1^*; *vas-Gal4 > GFP-Vas^GNT^* flies. Bars, 50 µm (egg chamber), 10 µm (nuage and pole plasm), and 100 µm (embryo).

Localization to nurse cell nuage was impaired in the case of GFP-Vas^DQAD^ (closed conformation) (Xiol *et al.* 2014), whereas GFP-Vas^WT^ and GFP-Vas^GNT^ (open conformation) showed wild-type localization (Figure 2, A and B). This was true for the localization of Aub and Ago3 (Figure S2, A and B and Figure S3A). These findings indicate that an open helicase conformation of the Vas is required for its correct localization, as well as for the localization of Aub and Ago3, whereas helicase activity per se is not.

**Figure 3:**
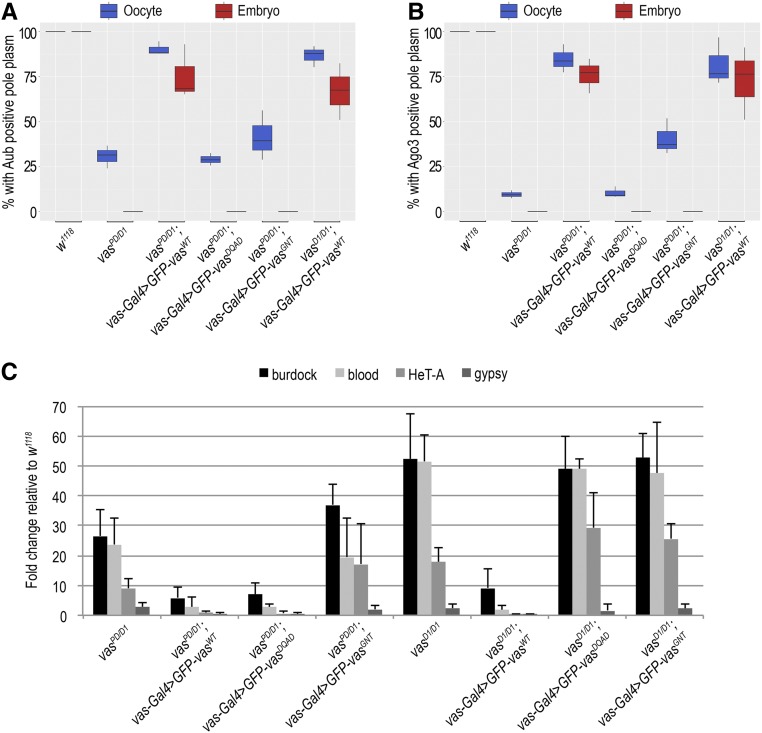
Localization of Aub and Ago3 in the egg chamber and embryos depends on Vasa. (A) Box plot showing percentage of oocytes and embryo progeny of wild-type (*w^1118^)*, *vas^PD/D1^*, *vas^PD/D1^*; *vas-Gal4 > GFP-Vas^WT^*, *vas^PD/D1^*; *vas-Gal4 > GFP-Vas^DQAD^*, *vas^PD/D1^*; *vas-Gal4 > GFP-Vas^GNT^*, and *vas^D1/D1^*; *vas-Gal4 > GFP-Vas^WT^* flies displaying Aub-positive pole plasm, as determined by immunohistochemical detection of Aub. Experiments were performed in three independent replicates. (B) Box plot representing percentage of oocytes and embryo progeny of wild-type (*w^1118^)*, *vas^PD/D1^*, *vas^PD/D1^*; *vas-Gal4 > GFP-Vas^WT^*, *vas^PD/D1^*; *vas-Gal4 > GFP-Vas^DQAD^*, *vas^PD/D1^*; *vas-Gal4 > GFP-Vas^GNT^*, and *vas^D1/D1^*; *vas-Gal4 > GFP-Vas^WT^* flies displaying Ago3-positive pole plasm, as determined by immunohistochemical detection of Ago3 protein. Experiments were performed in three independent replicates. (C) Quantitative PCR analysis for LTR transposons *burdock*, *blood*, and *gypsy* and non-LTR transposon *HeT-A* in *vas^PD/D1^*, *vas^PD/D1^*; *vas-Gal4 > GFP-Vas^WT^*, *vas^PD/D1^*; *vas-Gal4 > GFP-Vas^DQAD^*, *vas^PD/D1^*; *vas-Gal4 > GFP-Vas^GNT^*, *vas^PD/D1^*, *vas^PD/D1^*; *vas-Gal4 > GFP-Vas^WT^*, *vas^PD/D1^*; *vas-Gal4 > GFP-Vas^DQAD^*, and *vas^PD/D1^*; *vas-Gal4 > GFP-Vas^GNT^* flies. Expression of transposons in wild-type (*w^1118^*) was set to 1 and normalized to rp49 mRNA in individual experiments. Error bars represent SD from three biological replicates.

In oocytes and embryos, GFP-Vas^WT^ showed a wild-type localization at the posterior pole (Figure 2, A and B), whereas GFP-Vas^DQAD^ was not detected. Further, although we could detect GFP-Vas^GNT^ at the posterior pole of the oocyte and the protein was transmitted to the embryo, it was not detected at the posterior pole (Figure 2B and Figure S1E). In the presence of GFP-Vas^WT^ Aub and Ago3 showed wild-type localization in oocytes and embryos, whereas GFP-Vas^GNT^ only partially restored localization of the two PIWI proteins (Figure 3, A and B, Figure S2, C and D, and Figure S3, B–D). These observations indicate that helicase activity of Vas is necessary for stable localization of the protein itself and of Aub and Ago3 at the embryo posterior pole.

### Transposons are deregulated in flies expressing Vas helicase mutants

Interaction of Vas with PIWI proteins in the perinuclear nuage of nurse cells is required for PIWI-interacting RNA (piRNA) biogenesis and transposon control (Xiol *et al.* 2014; Nishida *et al.* 2015). To investigate the effects of Vas mutations on transposon control, we analyzed RNA levels of several transposons. Quantitative PCR analysis showed that expression of GFP-Vas^wt^ and GFP-Vas^DQAD^ but not GFP-Vas^GNT^ caused resilencing of transposons in *vas^PD/D1^* female germline (Figure 3C). GFP-Vas^DQAD^, previously described as a product-release-trap mutant (Xiol *et al.* 2014), entraps transposon RNAs within the piRNA amplifier complex, from which the cleaved transposon mRNAs cleaved cannot be released, as this requires ATP hydrolysis product release (Nishida *et al.* 2015). The entrapment of transposon RNAs can lead to transposon downregulation, as residual levels of endogenous Vas in *vas^PD/D1^* flies (Figure S1D) are sufficient to ensure that the piRNA amplification loop never collapses entirely. Our finding that only GFP-Vas^WT^, and not GFP-Vas^DQAD^ and GFP-Vas^GNT^, downregulated transposons in loss-of-function *vas^D1/D1^* flies (Figure 3C), suggests that the helicase activity of Vas is essential for transposon control.

### Vas-associated proteins in the *Drosophila* ovary

**Figure 4:**
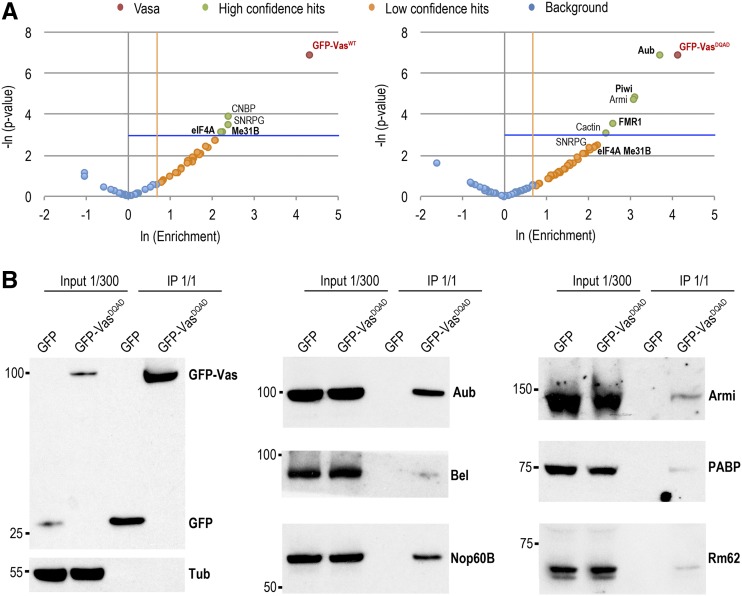
Vasa associates with proteins involved in early germ cell development . (A) Mass spectrometry analysis of GFP-Vas^WT^ (left panel) and GFP-Vas^DQAD^ (right panel) co-immunoprecipitations (co-IPs). Comparison of the fold-change in abundance of proteins based on spectral count ratio (enrichment >2-fold), and statistical significance (*P* < 0.05) of the fold-change between GFP-Vas co-IPs and GFP negative control co-IPs. Statistically significant proteins with the highest positive fold-change were considered high-confidence hits (green); proteins with a fold-change >2 but statistically nonsignificant (*P* > 0.05) were considered low-confidence hits (orange); proteins below both thresholds were considered background (blue). Vasa proteins are indicated in red. Proteins previously known to interact with Vasa are in bold. Statistical analysis was performed on two biological replicates. (B) Validation of Vas-interacting proteins identified by mass spectrometry presented in A. Western blot analyses were performed using antibodies against Armi, Aub, Bel, GFP, Nop60B, PABP, Rm62, and Tub showing amounts of the proteins present in the ovarian extracts (1/300 of the input) and recovered from the anti-GFP immunoprecipitations of GFP and GFP-Vas^DQAD^ (1/1 of the immunoprecipitation).

The dynamic association of DEAD-box RNA helicases with multiprotein complexes (Linder and Jankowsky 2011) renders challenging the biochemical detection of their interaction partners. The E400Q mutation, which locks Vas-containing protein complexes, is an ideal biochemical tool for identifying Vas’s interaction partners *in vivo* (Xiol *et al.* 2014). We performed co-immunoprecipitation experiments from ovaries expressing GFP-Vas^WT^ and GFP-Vas^DQAD^, and identified their associated proteins by mass spectrometry. Flies expressing GFP ubiquitously served as a negative control. We identified 57 proteins associated with GFP-Vas^WT^ and 71 associated with GFP-Vas^DQAD^ (Figure 4A, Figure S4, A–C, and Table S11). In the case of the GFP-Vas^WT^ co-immunoprecipitation, the stringent conditions and absence of a cross-linking reagent (see Materials and Methods) restricted detection to stable complexes. For instance, the Oskar protein, which interacts with Vas at the posterior pole (Breitwieser *et al.* 1996; Wang *et al.* 2015; Jeske *et al.* 2017), was not detected in the co-immunoprecipitations, whether in the case of GFP-Vas^WT^ or the “locked” GFP-Vas^DQAD^ (Figure 2B, top panels). We validated the specificity of co-immunoprecipitations by Western blot detection of several identified proteins. Our analysis confirmed Armi, Bel, PABP, Nop60B, and Rm62 as new Vas-associated proteins, while Aub served as a positive control (Figure 4B). Among the Vas interactors we identified were Aub, Piwi, Fragile X Mental Retardation 1 (FMR1), and eIF4A (Figure 4A), which have been shown to also be in complex with Vas in early embryos (Megosh *et al.* 2006; Thomson *et al.* 2008). Ago3 was not among the interactors, in agreement with previous findings that *Bombyx* Vas directly associates with Siwi (*Bombyx mori* Aub homolog) but not with Ago3 (Nishida *et al.* 2015). Curiously, we found CCHC-type zinc finger nucleic acid binding protein (CNBP) (Figure 4A), previously described to regulate wing development (Antonucci *et al.* 2014), to co-immunoprecipitate with Vas, suggesting that CNBP might also be involved in germline development. We also detected small ribonucleoprotein particle (Sm) proteins SmB, SmD2, SmD3, SmE, SNRPG, and snRNP-U1-70K to associate with Vas, leading to detection of splicing and spliceosomal complex as enriched gene ontology terms (Figure S4, D and E and Table S11). However, previous reports showed that Sm proteins localize at the posterior pole of the oocyte and that their localization is affected by Vas (Anne 2010; Gonsalvez *et al.* 2010), indicating involvement of Sm proteins in Vas-related cytoplasmic processes, rather than association of Vas with the nuclear process of splicing. Interestingly, the enrichment of proteins known to be involved in the female GSC divisions (Figure 4, A and B, Figure S4D, and Table S11), such as Aub, Rm62, PABP, FMR1, eIF4A, and Piwi, indicates a function of Vas early in oogenesis.

### Vas activity in the germarium is essential for oogenesis

**Figure 5:**
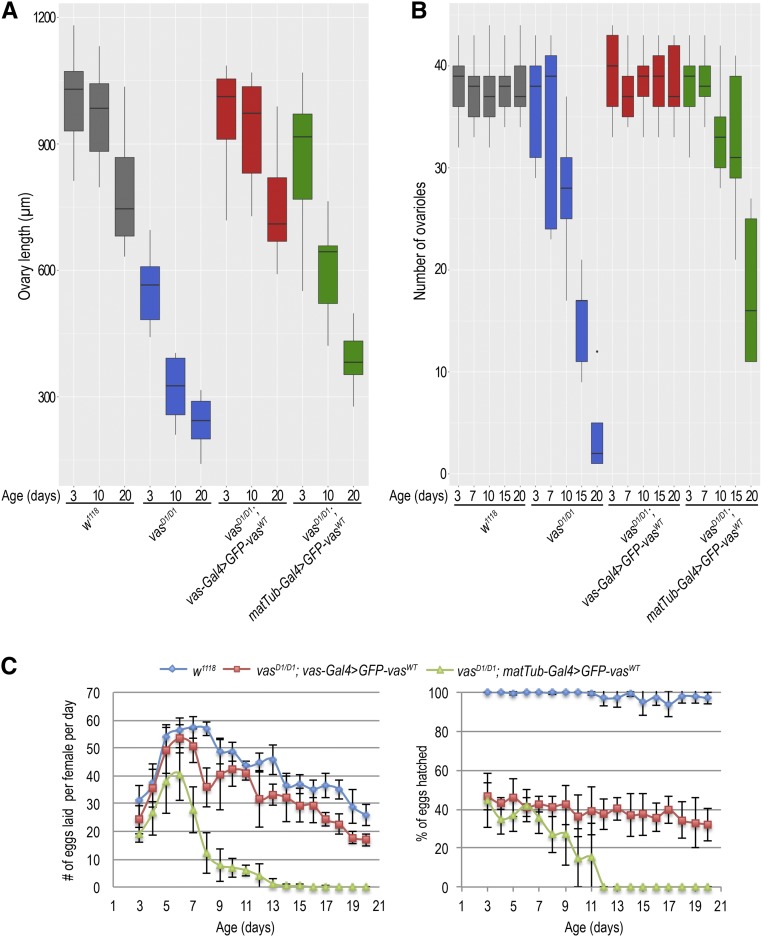
Vasa activity in the germarium is essential for germ cell development. (A) Box plot representing length of ovaries of 3-, 10-, and 20-day-old wild-type (*w^1118^)*, *vas^D1/D1^*, *vas^D1/D1^*; *vas-Gal4 > GFP-Vas^WT^*, and *vas^D1/D1^*; *matTub-Gal4 > GFP-Vas^WT^* flies. The measurements were performed on 10 flies (*n* = 10). (B) Box plot representing the number of egg chamber–producing ovarioles per 3-, 7-, 10-, 15-, and 20-day-old wild-type (*w^1118^)*, *vas^D1/D1^*, *vas^D1/D1^*; *vas-Gal4 > GFP-Vas^WT^*, and *vas^D1/D1^*; *matTub-Gal4 > GFP-Vas^WT^* females. Experiment was performed on five flies (*n* = 5). (C) Egg-laying rate (right panel) and hatching rate (left panel) measured daily between day 3 and day 20 postpupal enclosure of wild-type (*w^1118^)*, *vas^D1/D1^,* *vas^D1/D1^*; *vas-Gal4 > GFP-Vas^WT^*, and *vas^D1/D1^*; *matTub-Gal4 > GFP-Vas^WT^* females. Experiments were performed in five independent replicates.

Absence of Vas in *Drosophila* females causes oogenesis arrest (Lasko and Ashburner 1988, 1990). To determine at which stage of oogenesis Vas is required, we used either the *vas-Gal4* or the *matTub-Gal4* driver to express GFP-Vas^WT^ at distinct stages of oogenesis; the **vas** promoter is active throughout oogenesis, whereas the *matTub promoter* is inactive in the germarium, but active during the subsequent stages (Figure S1B). *matTub-Gal4* driven expression of GFP-Vas^WT^ in *vas^D1/D1^* females fully rescued oogenesis in 3-day-old flies, but as these progressed in age, oogenesis was arrested (Figure 5A and Figure S5A). In contrast, *vas-Gal4* driven expression of GFP-Vas^WT^ in the same *vas^D1/D1^* background restored oogenesis independently of the age of the flies (Figure 5A and Figure S5A). Of note, expression of helicase inactive GFP-Vas^DQAD^ and GFP-Vas^GNT^ proteins did not rescue oogenesis, regardless of the Gal4-driver used. In addition, analysis of egg chamber development showed that ovarian atrophy takes place between oogenesis stages six and eight and is a result of pyknosis (Figure S5B).

To test whether absence of Vas in the germarium interferes with germ cell development, we determined the number of egg chamber–producing ovarioles per female. Strikingly, in the case of *matTub-Gal4 > GFP-Vas^WT^* expressing *vas^D1/D1^* and *vas^D1/D1^* flies, the number of ovarioles decreased with the age of the females, whereas it did not in *vas-Gal4 > GFP-Vas^WT^* expressing *vas^D1/D1^* flies or in wild-type flies (Figure 5B). Furthermore, egg-laying analysis showed that the number of eggs produced by *vas^D1/D1^*; *matTub-Gal4 > GFP-Vas^WT^* females decreased with the age of the females and eventually stopped altogether (Figure 5C, left diagram). However, the hatching rate of eggs produced by *vas^D1/D1^* females as a result of *matTub-Gal4* or of *vas-Gal4 GFP-Vas^WT^* driven expression of *GFP-Vas^WT^* did not differ significantly (Figure 5C, right diagram). Taken together, these results indicate that progression and completion of oogenesis depends on the activity of Vas in the germarium.

### Absence of Vas in the germarium deregulates transposons in aged flies

**Figure 6:**
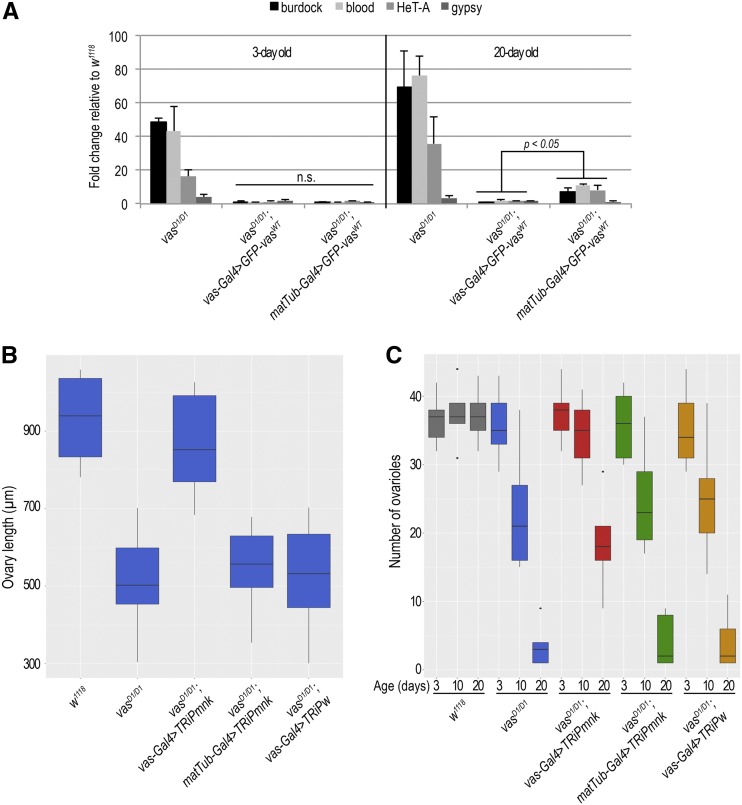
Chk2 signaling in the germarium induces arrest of germ cell development. (A) Quantitative PCR analysis for LTR transposons *burdock*, *blood*, and *gypsy* and non-LTR transposon *HeT-A* in 3- and 20-day-old *vas^D1/D1^*; *vas-Gal4 > GFP-Vas^WT^* and *vas^D1/D1^*; *matTub-Gal4 > GFP-Vas^WT^* flies. Expression of transposons in wild-type (*w^1118^*) was set to 1 and normalized to rp49 mRNA in individual experiments. Error bars represent SD from two biological replicates. *P*-values were determined by Student’s *t*-test. (B) Box plot representing length of ovaries of wild-type (*w^1118^)*, *vas^D1/D1^*, *vas^D1/D1^*; *vas-Gal4 > TRiPmnk*, *vas^D1/D1^*; *matTub-Gal4 > TRiPmnk*, and *vas^D1/D1^*; *vas-Gal4 > TRiPw* flies. The measurements were performed on 15 flies (*n* = 15). (C) Box plot representing the number of egg chamber–producing ovarioles per 3-, 10-, and 20-day-old wild-type (*w^1118^)*, *vas^D1/D1^*, *vas^D1/D1^*; *vas-Gal4 > TRiPmnk*, *vas^D1/D1^*; *matTub-Gal4 > TRiPmnk*, and *vas*^D1/D1^; *vas-Gal4 > TRiPw* females. Experiment was performed on five flies (*n* = 5). Dots in the box plot represent values that are 1.5 times greater than the upper limit or 1.5 time smaller than the lower limit of the interquartile range.

As removal of Vas causes transposon upregulation (Liang *et al.* 1994; Malone *et al.* 2009), we tested whether oogenesis arrest in *vas^D1/D1^*; *matTub-Gal4 > GFP-Vas^WT^* females coincides with transposon deregulation. Expression of *GFP-Vas^WT^* using the *matTub-Gal4* or *vas-Gal4* drivers silenced transposons to similar levels in *vas^D1/D1^* 3-day-old flies (Figure 6A). In aged 20-day-old females, however, we could observe small but significant increase of transposon mRNA levels in *vas^D1/D1^* flies expressing *matTub-Gal4 > GFP-Vas^WT^* compared to *vas-Gal4 > GFP-Vas^WT^* (Figure 6A). These results suggest that absence of Vas in the germarium leads to a delayed transposon deregulation that coincides with the arrest of germline development.

### Chk2 signaling in the germarium induces oogenesis arrest in **vas** mutant *Drosophila*

We recently showed that *mnk* (Chk2) and **vas** interact genetically, and that depletion of Chk2 signaling in loss-of-function *vas^D1/D1^* flies rescues oogenesis, but that the embryos die due to severe DNA damage (Durdevic *et al.* 2018). Moreover, previous studies determined that Vas is phosphorylated in a Chk2-dependent manner (Abdu *et al.* 2002; Klattenhoff *et al.* 2007). To investigate whether the age-dependent oogenesis arrest observed in *vas^D1/D1^*; *matTub-Gal4 > GFP-Vas^WT^* flies is due to the Chk2 signaling, we used RNA interference (RNAi) to knockdown *mnk* mRNA by either *matTub-Gal4* or *vas-Gal4* driven expression of *mnk* double-strand RNA (*vas^D1/D1^*; *matTub-Gal4 > TRiPmnk* and *vas^D1/D1^*; *vas-Gal4 > TRiPmnk*, respectively). Knockdown efficiency tests using quantitative PCR showed *mnk* mRNA levels to be between 30 and 40% of the wild-type *mnk* level (Figure S6A). However, fluorescence *in situ* RNA hybridization analysis showed that upon *matTub-Gal4* driven knockdown of *mnk*, the mRNA was detectable in the germarium and not in the later stages of oogenesis, whereas *vas-Gal4* driven *mnk*-RNAi downregulated *mnk* throughout oogenesis (Figure S6B). Furthermore, although *vas-Gal4* driven silencing of *mnk* in *vas^D1/D1^* females restored oogenesis, *matTub-Gal4* driven knockdown of *mnk* did not (Figure 6B). This indicates Chk2-mediated signaling activity in the germarium determines the fate of developing egg chambers. Although the efficiency of *vas**-Gal4* RNAi-driven downregulation of *mnk* decreased over time, finally resulting in ovarian atrophy, we observed a more severe age-dependent decrease in the number of egg chamber–producing ovarioles when *mnk* knockdown was driven by *matTub-Gal4* (Figure 6C). These results suggest that in **vas** mutants Chk2 signaling specifically in the germarium induces arrest of germ cell development.

## Discussion

We have demonstrated that development of the *Drosophila* female germline depends on Vas activity in early oogenesis. Our data indicate that progression and completion of oogenesis require helicase active Vas. However, as our fusion proteins show low-expression levels, we cannot rule out that when expressed at higher levels Vas might support oogenesis independently of helicase activity (Dehghani and Lasko 2015). Independent of helicase activity is Vas’s subcellular localization, which requires an open conformation of the protein. Helicase mutant GFP-Vas^DQAD^, which is unable to release the ATP-hydrolysis products (Xiol *et al.* 2014), shows strong association with piRNA pathway components and colocalizes with Aub and Ago3 in large foci in the nurse cells. The granular accumulation of GFP-Vas^DQAD^ presumably hinders localization of the helicase to the posterior pole of the oocyte. In contrast, helicase inactive mutant GFP-Vas^GNT^, which remains in an open conformation, displays both wild-type localization and correct subcellular distribution. However, Vas blocked in an open conformation does not maintain its posterior accumulation in the embryos. Thus, we show that localization of Vas in the egg chambers, but not in the embryos, is helicase activity independent and requires open conformation of the protein.

Vas interaction with different factors implicated in promoting the GSC division indicates an intricate network of Vas-associated processes involved in sustaining the germ cell lineage. Moreover, the previously overlooked fact that loss-of-function **vas** mutant flies undergo an age-dependent reduction of the ovariole number indicates Vas function in early germ cell development. Using stage-specific promoters, we manipulated the expression of Vas and determined that activity of Vas in the germarium is crucial for sustaining germ cell lineage. Our conclusion that oogenesis depends on an early helicase activity of Vas is consistent with the finding that Vas directly interacts with *meiotic P26* (mei-P26*)* mRNA and activates its translation (Liu *et al.* 2009). Mei-P26 itself has been found to cooperate with proteins such as Bag of marbles and Sex lethal to promote both GSC self-renewal and germline differentiation (Li *et al.* 2012, 2013). In addition, we identified Vas interaction partners Lingerer, Rasputin, FMR1, and Caprin, which have been shown to cooperate in restricting tissue growth in a nongermline tissue, the *Drosophila* eye (Baumgartner *et al.* 2013). Interestingly, these proteins were found to interact in *Drosophila* ovaries as well (Costa *et al.* 2005, 2013), suggesting a complex that could act in conjunction with Vas to control growth of *Drosophila* germline tissue. FMR1, Piwi, and Aub were previously shown to interact with Vas in embryos and to be important for primordial germ cell formation (Deshpande *et al.* 2006; Megosh *et al.* 2006). In *Drosophila* ovaries, FMR1 was proposed to participate in the regulation of germline proliferation and GSC maintenance (Yang *et al.* 2007; Epstein *et al.* 2009), while Piwi and Aub have been shown to control GSC self-renewal and lineage differentiation (Cox *et al.* 1998; Ma *et al.* 2014, 2017). Vas association with Rm62, PABP, and eIF4A suggests that Vas might be a part of a previously described interaction network of Aub, eIF4, PABP, and Rm62 that regulates germ cell lineage development (Ma *et al.* 2017). We have also shown that Vas associates with Bel and Nop60B, proteins that play a role in male GSC maintenance (Kauffman *et al.* 2003; Kotov *et al.* 2016). Our study thus reveals association of Vas with different factors involved in the control of stem cell proliferation and maintenance. Further studies will be required to determine how these proteins collaborate to regulate early germ cell development.

In *Drosophila*, Chk2 signaling triggered by DNA damage, replication stress, or nuclear lamina dysfunction induces GSC loss (Molla-Herman *et al.* 2015; Ma *et al.* 2016; Barton *et al.* 2018). Our previous study showed that removal of Chk2 in **vas** mutant flies fully restores oogenesis, while progeny embryos succumb to transposon upregulation and DNA damage (Durdevic *et al.* 2018). Here, we went further and genetically determined that, in **vas** mutants, Chk2 signaling exclusively in the germarium is sufficient to cause oogenesis arrest. Furthermore, the decline of ovariole number is Chk2-dependent, indicating that in **vas** mutants Chk2 signaling compromises germ cell lineage proliferation. An earlier study suggested that Vas interacts with Aub to regulate mitotic chromosome condensation in *Drosophila* GSCs and that consequently, **vas** mutants display aberrant chromosome segregation during GSC mitosis (Pek and Kai 2011b). Defects in mitotic chromosome segregation can be the cause as well as the consequence of Chk2 activation in the respective daughter cells (Janssen *et al.* 2011; Bakhoum *et al.* 2014). As **vas** mutant GSCs do not display DNA damage (Pek and Kai 2011b), we speculate that mitotic chromosome segregation defects trigger Chk2 signaling in the daughter GSC and the CB, disrupting GSC self-renewal and development of the germ cell lineage. We conclude that the activity of Vas RNA helicase in germarium is critical to ensure sustained development of the germ cell lineage in *Drosophila*.
